# Prenatal alcohol exposure is associated with changes in placental gene co-expression networks

**DOI:** 10.1038/s41598-024-52737-6

**Published:** 2024-02-01

**Authors:** Maya A. Deyssenroth, Randy P. Williams, Corina Lesseur, Sandra W. Jacobson, Joseph L. Jacobson, Haoxiang Cheng, Promita Bose, Qian Li, Helen Wainwright, Ernesta M. Meintjes, Ke Hao, Jia Chen, R. Colin Carter

**Affiliations:** 1 Department of Environmental Health Sciences, Mailman School of Public Health, Columbia University, New York, NY USA; 2 Department of Environmental Medicine and Public Health, Icahn School of Medicine at Mount Sinai, New York, NY USA; 3 Department of Psychiatry and Behavioral Neurosciences, Wayne State University School of Medicine, Detroit, MI USA; 4 Department of Human Biology, University of Cape Town Faculty of Health Sciences, Cape Town, South Africa; 5 Department of Psychiatry and Mental Health, Faculty of Health Sciences, University of Cape Town, Cape Town, South Africa; 6 Department of Genetics and Genomic Sciences, Icahn School of Medicine at Mount Sinai, New York, NY USA; 7 Department of Pathology, National Health Laboratory Service, Cape Town, South Africa; 8 Departments of Emergency Medicine and Pediatrics, Institute of Human Nutrition, Columbia University Vagelos College of Physicians and Surgeons, New York, NY USA

**Keywords:** Developmental biology, Biomarkers

## Abstract

Alcohol consumption during pregnancy can result in a range of adverse postnatal outcomes among exposed children. However, identifying at-risk children is challenging given the difficulty to confirm prenatal alcohol exposure and the lack of early diagnostic tools. Placental surveys present an important opportunity to uncover early biomarkers to identify those at risk. Here, we report the first transcriptome-wide evaluation to comprehensively evaluate human placental pathways altered by fetal alcohol exposure. In a prospective longitudinal birth cohort in Cape Town, South Africa, we performed bulk tissue RNAseq in placenta samples from 32 women reporting heavy drinking during pregnancy and 30 abstainers/light drinkers. Weighted gene co-expression network analysis (WGCNA) and differential gene expression analysis were performed to assess associations between fetal alcohol exposure and placental gene expression patterns at a network-wide and single gene level, respectively. The results revealed altered expression in genes related to erythropoiesis and angiogenesis, which are implicated in established postnatal phenotypes related to alcohol exposure, including disruptions in iron homeostasis, growth, and neurodevelopment. The reported findings provide insights into the molecular pathways affected by prenatal alcohol exposure and highlight the potential of placental biomarkers for detecting and understanding the effects of alcohol on fetal development.

## Introduction

Fetal alcohol spectrum disorders (FASD) constitute a range of deleterious outcomes that can occur in children as a consequence of maternal alcohol consumption during pregnancy. While the teratogenic effects of alcohol exposure are well established^1^, FASD continue to persist. In the US, FASD prevalence is estimated to range between 2 and 5% among US school-age children^2–4^. In addition to the burden to the individual with respect to academic achievement and neurodevelopmental and behavioral pathology, there are also considerable societal costs in terms of required mental health services, substance use treatment centers, long-term care services, and interactions with the criminal justice system^5^. As one of the most preventable causes of neurodevelopmental deficits, prevention as well as early intervention are key to mitigating these far-reaching consequences.

Identifying children at-risk for developing FASD is challenging. For one, establishing the presence and magnitude of prenatal alcohol exposure (PAE) is difficult to confirm through maternal self-report due to the associated stigma^6^. While there are well-characterized facial dysmorphology features specific to PAE, most children do not display the full pattern of physical signs. Indeed, determination of FASD often does not occur until school age, when learning and behavioral problems become first reliably apparent^7^. Given that the most effective timing for intervention is during infancy, this lag in identification likely limits the ability to realize the full scope of intervention. In addition, there is considerable overlap between the cognitive and behavioral deficits in FASD that arise in childhood and those associated with other etiologies, such as ADHD, complicating mechanistic studies seeking to determine the specific role of PAE on these latent effects. There is currently no universally accepted diagnostic tool applicable in early life to address these concerns. Perinatal biomarkers offer the means to develop sensitive and specific screening tools that identify neonates vulnerable to develop the effects associated with PAE.

The placenta is ideally suited to develop early screening biomarkers of PAE. This organ exists during the developmental window of interest that spans from implantation to parturition, captures the cross-talk between the fetal and maternal compartment, reflects gestational quality, and is non-invasively accessible. The potential relevance of this organ in the FASD paradigm is highlighted by various adverse placental outcomes shown to be associated with PAE^8^, including placental accreta^9^, placental abruption^9–12^, reduced placental weight^13–15^, and altered placental vasculature^16–21^. Importantly, these placental defects are also known to impede appropriate fetal growth and development and suggest that placental biomarkers may reveal critical mechanistic insights underlying FASD.

A number of studies have examined placental molecular profiles to identify developmental FASD biomarkers. Animal studies have revealed alcohol-responsive changes in candidate protein markers^22,23^ and gene expression^24,25^. Findings from human epidemiologic studies to date have similarly reported on specific candidate pathways of interest, including lower iodine storage^26^, fatty acid ethyl ester levels^27^, levels of proteins participating in angiogenesis- and pro-inflammatory cytokine pathways^28^, and changes in imprinted gene expression patterns^29^. Studies examining a range of other prenatal exposures have also showcased the promise of profiling gene expression patterns in the placenta to provide insight into altered developmental pathways. For example, several placental gene expression profile studies have highlighted the downregulation of genes involved in the synthesis and transport of serotonin, a critical component of early brain development, in response to bisphenol A^30^, valproic acid^31^ and flame retardants. These studies suggest that placenta gene expression profiles can reveal exposure-induced pathways with important clinical implication for early intervention. Here, we conducted a transcriptome-wide survey of placental tissue samples with and without heavy prenatal alcohol exposure from a prospective birth cohort in Cape Town, South Africa, to comprehensively evaluate alcohol-responsive placental molecular pathways.

## Results

### Study population characteristics

Characteristics of the study population are shown in Table 1. Individuals who drank during pregnancy did not differ from pregnant individuals who largely abstained from drinking based on gestational age at delivery, birth weight, infant sex, maternal parity, or maternal education. However, the average maternal age of exposed participants was higher than unexposed participants.

**Table 1:** Participant characteristics by prenatal alcohol exposure status.

Variable	Exposed	Unexposed	*p* value^b^
	*N* = 33 (53.2%)^a^	*N* = 29 (46.8%)^a^	
Maternal age (years)	29.66 (5.89)	24.60 (4.76)	< 0.001
Maternal education (years)			0.91
< 10th grade	13 (39.4%)	11 (37.9%)	
At least 10th grade	20 (60.6%)	18 (62.1%)	
Gestational age at delivery (weeks)	39.11 (1.84)	39.39 (1.61)	0.42
Birth weight (grams)	2,932.12 (530.91)	3,124.83 (517.79)	0.19
Infant sex			0.69
Female	12 (36.4%)	12 (41.4%)	
Male	21 (63.6%)	17 (58.6%)	
Parity			0.17
Nulliparous	3 (9.1%)	7 (24.1%)	
Parous	30 (90.9%)	22 (75.9%)	
Avg. daily alcohol (oz)	0.82 (0.65)	0.00 (0.01)	< 0.001

^a^Mean (SD); n (%).

^b^Wilcoxon rank sum test; Pearson's Chi-squared test; Fisher's exact test.

### Differential gene expression analysis

**Figure 1:**
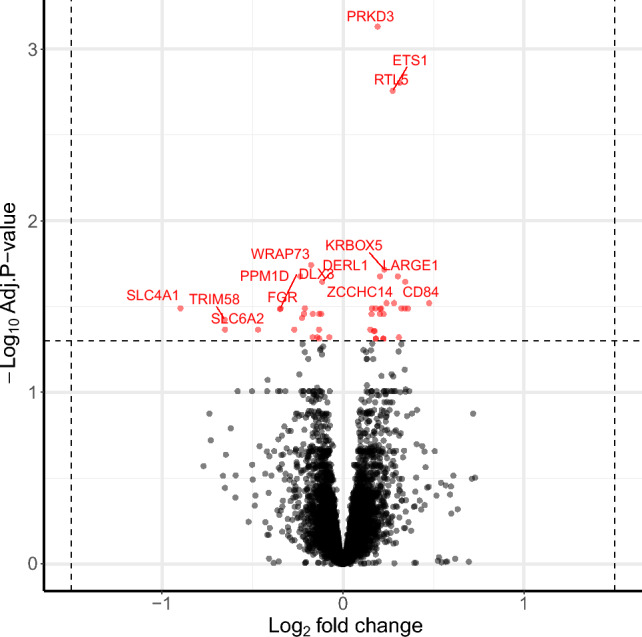
Differentially expressed genes in placental samples based on alcohol exposure across pregnancy. Models were adjusted for 7 surrogate variables, gestational age, infant sex, maternal age, and cell type proportions**.**

Using an FDR < 0.05 cutoff, we observed 40 genes that were differentially expressed by PAE status (Fig. 1; Supplemental Table 1).

### Weighted gene co-expression analysis

**Figure 2:**
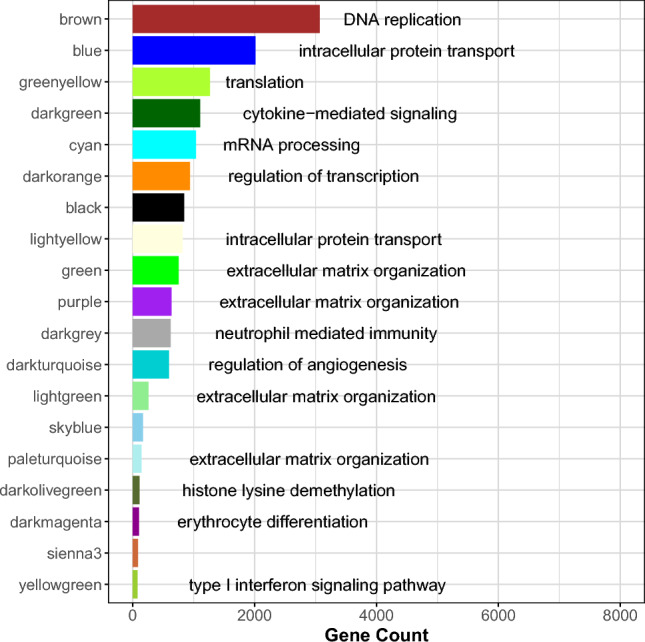
Gene ontology enrichment of placental coexpression network. Gene counts (x-axis) for the 19 identified modules (y-axis) are shown along with associated gene ontology processes. Several modules (darkmagenta, tan, steelblue, skyblue3, and grey) were not enriched for specific processes.

Using weighted gene co-expression network analysis, we identified 19 network modules, shown in Fig. 2 with their associated gene ontologies. Module processes identified in this network align with previously published placental co-expression networks, in which enriched processes also include immune-related pathways, epigenetic regulation, cellular replication, metabolic processes, extracellular matrix organization, and angiogenesis^32,33^. The identified modules were associated with several demographic characteristics (Supplemental Fig. 1). These include gestational age (neutrophil mediated immunity [darkgrey] and cytokine-mediated signaling [darkgreen]), birth weight (extracellular matrix organization [paleturquoise]), maternal age (neutrophil mediated immunity [darkgrey]), sex (mRNA processing [cyan], histone lysine demethylation [darkolivegreen]), parity (neutrophil mediated immunity [darkgrey], regulation of transcription [darkorange], regulation of angiogenesis [darkturquoise], extracellular matrix organization [lightgreen]), and maternal education (black, skyblue). Several modules also varied by cell type composition. For example, the three immune-related modules, darkgreen, yellowgreen and darkgrey, correlated with changes in the proportion of Hofbauer cells, the predominant placental macrophages. Similarly, the angiogenesis-related darkturquoise module correlated with endothelial cell proportion.

**Figure 3:**
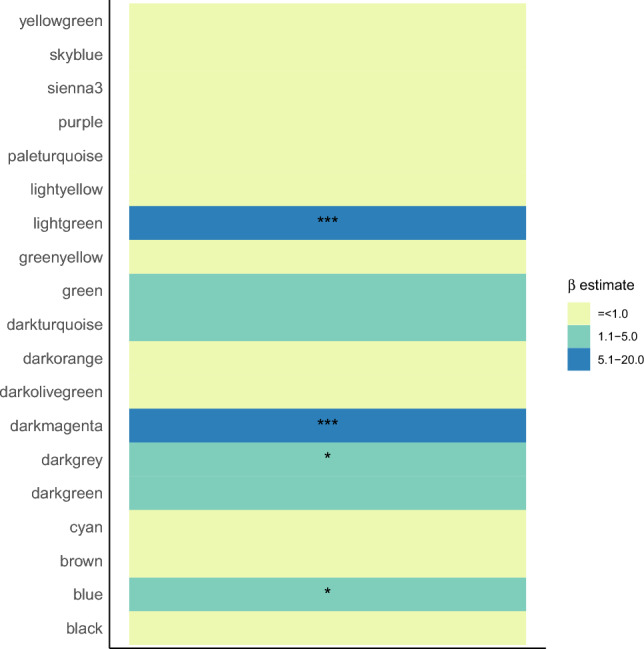
Fisher exact test enrichment of genes differentially expressed by prenatal alcohol exposure status among placental gene coexpression modules. Asterisks indicate modules that are significantly enriched with differentially expressed genes at FDR-corrected p values < 0.05 (***), < 0.1 (**), and < 0.2 (*). The magnitude of the enrichment is indicated by the color, with the scale spanning from negative enrichment (yellow) to positive enrichment (blue).

We observed significant, positive enrichment of genes differentially expressed by PAE status in several modules (Fig. 3). These included modules involved in extracellular matrix organization [lightgreen], erythrocyte differentiation [darkmagenta], neutrophil mediated immunity [darkgrey], and intracellular protein transport [blue].

**Figure 4:**
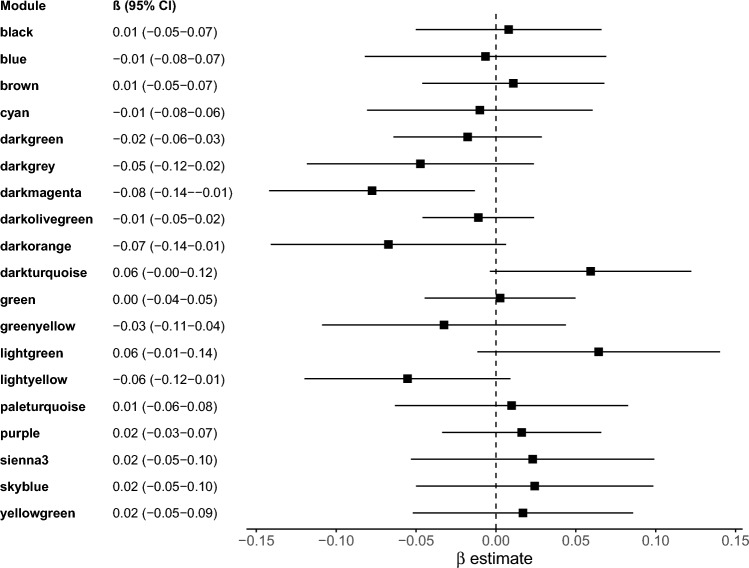
Placental coexpression modules associated with prenatal alcohol exposure. The forest plot depicts β estimates and 95% confidence intervals from linear regression models assessing the association between module eigengene values and alcohol exposure status, adjusting for potential confounders.

Examining continuous measures of PAE, oz. of alcohol consumed per occasion was positively correlated with regulation of angiogenesis (darkturquoise) and negatively correlated with neutrophil mediated immunity (darkgrey) (Supplemental Fig. 1A). Examining PAE as a binary variable, mean co-expression of the erythrocyte differentiation (darkmagenta) module was reduced and mean co-expression of the regulation of angiogenesis (darkturquoise) and extracellular matrix organization (lightgreen) modules was elevated in exposed compared to unexposed participants. In multivariable regression modules examining associations between module eigengenes and PAE status, adjusting for surrogate variables, maternal age, gestational age, infant sex, and cell type composition (Fig. 4), PAE was associated with downregulated co-expression for the erythrocyte differentiation (darkmagenta) module. Upregulation of the angiogenesis (darkturquoise) module was also observed at borderline significance.

**Figure 5:**
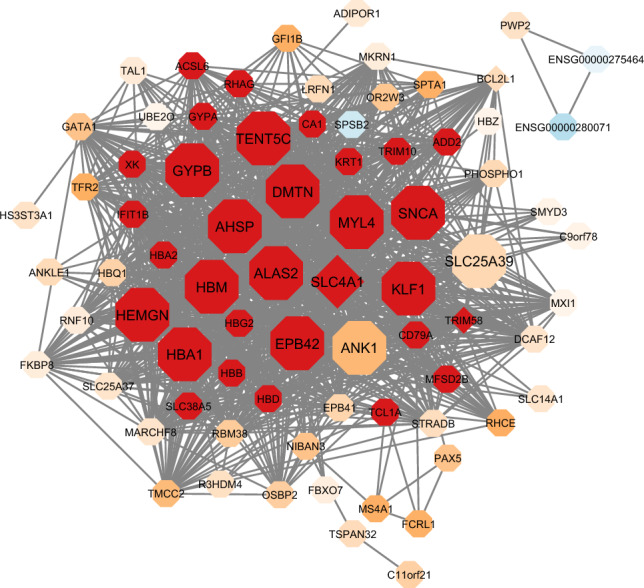
Connectivity map of darkmagenta module. Differentially expressed genes based on PAE status are indicated as diamond-shaped nodes. The color of the nodes indicates the direction of the association with alcohol exposure, ranging from positive fold-change values (blue) to negative fold-change values (red). Hub genes are indicated as larger sized nodes.

As shown in the connectivity map of the erythrocyte differentiation (darkmagenta) module in Fig. 5, there was a general downregulation across the genes in this module in association with PAE, particularly among the hub genes. Among genes differentially expressed by PAE status, 3 genes (*TRIM58*, *BCL2L1* and *SLC4A1*) loaded onto this module.

## Discussion

Our analysis highlights placental expression changes in specific genes as well as coregulated gene-sets in association with PAE that may be used to inform future biomarkers of exposure and effect.

These findings are based on both differential gene expression analysis and network-based co-regulated gene-sets. Each approach has its own as well as complementary utility in candidate biomarker discovery. Differential gene expression analysis identifies a specific set of individual loci. However, as each gene is examined independently, only signals with effect sizes large enough to pass FDR-screening are usually detected; this may lead to false negative findings for genes with smaller effect sizes that may collectively have important predictive and/or mechanistic relevance. Furthermore, this approach may be vulnerable to over-fitting for a given study cohort, and individual gene hits found in one cohort may be difficult to replicate in other populations. Network analysis examines coregulation among groups of genes that participate in common biological pathways and thus identifies differentially expressed networks that comprise pathways or mechanistic themes rather than individual genes. Since module associations are based on many genes rather than a few large gene signals, results may be more generalizable outside of the original study cohort. A critical next step in biomarker methodology is the practical creation of biomarker-based classifiers to identify individuals with exposure and/or exposure-related effects of clinical interest. To our knowledge, it is unclear whether such classifiers would perform better if based on candidate biomarkers from differential gene expression versus network-based approaches; this question comprises a promising direction for future research.

Convergence between these two approaches may aid in prioritizing individual loci based on their involvement in biologically meaningful pathways. In our analysis, several genes were differentially expressed and also mapped to differentially co-expressed modules. The erythrocyte differentiation (darkmagenta) module was significantly downregulated in association with PAE, and significantly enriched for genes that were also downregulated among exposed compared to unexposed participants based on differential gene expression analysis (*BCL2L1*, *TRIM58, and SLC4A1*). *SLC4A1* is additionally a hub gene within the module (i.e., within top 15 genes based on intramodular connectivity scores). The darkturquoise module (regulation of angiogenesis) was upregulated at borderline significance in association to PAE status, and included genes that were upregulated among exposed compared to unexposed participants based on differentially gene expression analysis (*ETS1* and *EGLN1*). Although the lightgreen module (extracellular matrix organization) was not related to PAE, it was significantly enriched for genes that were differentially expressed by PAE status (*RTL5, BHLHE41, TRIM2, ASCC2, PDGFRB*). Several of these observations are consistent with previously published experimental studies that show an association between ethanol exposure and decreased hepatic *BCL2L1* expression in exposed rats^34^, altered synaptic splicing in ethanol-exposed mice for *TRIM2*^35^, *EGLN1*^35^, and *ETS1*^35^ as well as overall *EGLN1*^36^ upregulation in ethanol exposed embryoid bodies, and sex-specific reductions in *PDGFRB*^37^ at the blood–brain barrier of ethanol-exposed adolescent rats.

Literature support also exists for genes in our study that were differentially expressed by PAE status but did not map to differentially co-expressed modules. These include genes that mapped to the intracellular protein transport (blue) module (*DERL1*^38^, *KMT2A*^35,39^, *MAP3K1*^40^,*PRKD3*^35,41^, *ZCCHC14*^35^), the cytokine-mediated signaling (darkgreen) module (*BMP2K*^35^, *CD84*^35^), and the DNA replication (brown) module (*MEF2A*^42^, *RER1*^38^). Similar support also exists for several hub genes of differentially co-expressed modules that did not demonstrate differential gene expression by PAE status in our study. These include erythrocyte differentiation (darkmagenta) module hub genes (*ALAS2*^43^, *DMTN*^35^, *ANK1*^35^, *SNCA*^35,44, 45^, and *SLC25A39*^38^) and the regulation of angiogenesis (darkturquoise) hub genes (*KDR*^46^, *SCARB1*^47^). Our findings highlight that genes sensitive to alcohol exposure in experimental settings across the life course and tissue contexts (e.g., liver and brain) are also sensitive to developmental alcohol exposure based on differential placental gene activity in a human observational study.

Our primary findings suggest that erythropoiesis-related activity in the placenta is sensitive to PAE. These findings are in line with a recent mouse model study that also observed changes in placental expression of erythrocyte differentiation genes in response to alcohol exposure^48^. The origin of the altered erythropoiesis signals remains unclear. In our data, mature erythrocytes accounted for less than 1% of the median estimated cell type proportion, displayed low inter-sample variability, and were fully absent in 64% of samples^49^. Due to their negligible estimated cell counts in our data, our cell-type adjusted models did not include erythrocyte proportions. It is possible that we are either underestimating the presence of erythrocytes in our data or lack the resolution to identify changes in low count cell types. Therefore, we cannot rule out that the differential signals we detect are not at least partially a reflection of a reduction in erythrocyte cells among exposed placentas. Alternatively, the erythropoietic signals we detected may reflect activities of the placenta itself as a source of fetal hematopoiesis, an increasingly recognized role early in gestation^50^. As a hematopoietic center, the placenta generates differentiated blood cells for immediate access early in embryonic development and a pool of undifferentiated stem cells as a long-term postnatal resource^51^. To date, we and others have reported effects of PAE on maternal and fetal hemoglobin synthesis, resulting iron deficiency^52–54^, and potential links to FASD-related neurobehavioral deficits^55^.

Our data also suggest additional placental pathways sensitive to PAE. Of note, angiogenesis regulation (darkturquoise) is a well substantiated pathway identified in animal models tracing the effects of PAE^46^. Several studies have reported on alcohol-induced alterations in vascular endothelial growth factor (VEGF)-mediated angiogenesis^56–58^, a critical component of placental vascularization that is necessary for adequate nutrient and gas exchange. Disruption of this process is posited to underly placental insufficiency and fetal growth restriction.

Several limitations in our study warrant consideration. Our findings stem from data from the Cape Coloured community, a community where prenatal alcohol consumption is likely driven by underlying factors and norms unique to this population^59^. These include remnants from oppressive colonial labor practices, in which farm workers were compensated for their labor using alcohol, normalizing excessive consumption. Historically inequitable access to quality maternal health care also compromises access to care and critical gateways to communicate risk effectively. The unique presentation of prenatal alcohol consumption coupled with the small sample size of our study may hinder the generalizability of our findings. Replication in a larger cohort is needed to verify the robustness of our report. However, the distinguishing aspects of this population also serve as strengths. In a setting where hazardous drinking during pregnancy is common, i.e., exposure levels are especially high, and drug co-exposures are relatively uncommon, there is enhanced power to detect differences across exposure profiles despite the small sample size of our study. The high rate of FASD in this setting also highlights the importance to characterize biomarkers relevant to this underserved, at-risk population. Finally, given the constraints in our sample size, we also opted to maximize our power to detect relevant loci by restricting our genome-wide survey to protein-coding regions. A noteworthy drawback of this approach is that we excluded important regulatory loci (e.g., miRNA) from our analysis. This drawback is particularly relevant for our differential gene expression analysis. However, given the known regulatory relationship between coding and non-coding transcripts, we expect that in a well-powered study that could accommodate the full scope of transcribed loci, the inclusion of non-coding loci would likely reinforce the coregulated patterns detected in our protein-coding networks.

This study builds on prior research that has highlighted the potential of the placenta, a developmentally relevant, yet understudied, target tissue, to provide mechanistic insight into early determinants of PAE. Our findings in a human population study coincide with reported findings from experimental studies. Importantly, the alcohol-responsive molecular pathways that we identified, including erythropoiesis and angiogenesis have known implications for established postnatal phenotypes related to alcohol exposure, such as PAE-related disruptions in iron homeostasis, growth, and neurodevelopment. These findings, therefore, suggest potential relevance for these placental biomarkers not only as indicators of exposure but also as possible candidate markers of effect.

## Methods

### Study participants

During their first antenatal visit at one of two midwife obstetric units that serve the Cape Coloured community in Cape Town, South Africa, pregnant women were enrolled in a prospective, longitudinal birth cohort examining the long-term effects of PAE on child development^19,60^. At the enrollment interview, participants were asked about drinking habits during the past 2 weeks and during a typical 2-week period around the time of conception. Recruitment focused on individuals who reported drinking on average at least 1 oz absolute alcohol (AA)/day (~ 1.67 standard drinks) or binge drinking (≥ 2 oz AA/drinking occasion) on at least three occasions. Individuals who reported that they abstained from drinking or drank minimally (no binge episodes) were invited to participate as controls. Individuals were eligible to participate in the parent study if they were at least 18 years old, carrying singleton pregnancies, HIV-negative, and not undergoing pharmacologic treatment for medical conditions. For the sub-study presented here, additional exclusion criteria included regular methamphetamine use (at least monthly), maternal medical conditions known to affect placental development (hypertension, preeclampsia, and syphilis), and delivery before 34 weeks gestation. Informed consent, conducted in the participant’s preferred language (Afrikaans or English) was obtained from all participants. Study protocol approval was obtained from the Institutional Review Boards at Wayne State University, the University of Cape Town Faculty of Health Sciences, and Columbia University Irving Medical Center, and all research was performed in accordance with relevant regulations.

### PAE

At recruitment and again at 4 and 12 weeks into study participation, individuals were interviewed about their drinking habits, including type and amount of each beverage, on a day-by-day basis during the previous 2 weeks as well as the 3-week period around the time of conception, with recall linked to specific times of daily activities in timeline follow-back interviews^6,61^. Participants were also interviewed regarding cigarette and drug (cocaine, methamphetamine, opiates, methaqualone, and marijuana) use; urine ELISA drug testing was used to validate interviews^19^. These responses were used to calculate the following summary measures averaged across pregnancy: oz absolute alcohol (AA)/day (1.0 oz = 30 mL = 1.67 standard drinks), oz AA/drinking occasion, and frequency of drinking. These summary measures were also dichotomized to generate a binary PAE variable. Participants were assigned PAE status if they reported drinking a daily average of at least 1.0 oz AA/day and/or binge drinking (≥ 2.0 oz AA per drinking occasion). All remaining participants reporting light-to-no exposure were considered unexposed.

### Placenta collection and RNA extraction

Placentas were stored at 2 °C immediately following delivery. Biopsies free of maternal decidua were excised from four quadrants within 2 cm of the cord insertion site and flash-frozen at − 80 °C within 72 h of delivery^29^. Total RNA was isolated from homogenized placental tissue from frozen samples at a later date using the Maxwell 16 LEV simplyRNA Tissue Kit (Promega, #AS1280; Madison, WI). RNA yield was quantified using a Nanodrop Spectrophotometer (Thermo Fisher Scientific, #ND-2000; Waltham, MA), and RNA integrity was measured using an Agilent Bioanalyzer (Agilent Technologies, Santa Clara, CA).

### RNA sequencing

RNA from 69 individual placental samples and 3 replicates were submitted for RNA sequencing. One hundred base pair reads were generated at 50 million reads per sample. Raw reads were trimmed to remove adaptor sequences and filtered to remove low quality reads based on Phred score values < 20. Gene-level abundances were quantitated based on the GRCh38.v33 human reference genome using STAR. The data were restricted to genes with expression counts > 10 in a minimum of 50% of the samples. Detected genes were further restricted to protein-coding genes. The final dataset included 14,867 genes. Based on a prior study that integrated infant and maternal gene expression and genotyping data^49^, one sample with > 5% estimated maternal cell contamination was identified and removed from the current study. Samples with RIN < 4 (*n* = 4) were also excluded from the analysis. The final sample included 62 participants.

### Statistical analysis

We performed surrogate variable analysis using the sva R package^62^ and identified 7 surrogate variables that represent hidden confounding variables in our data. In a prior analysis^49^ that leveraged an existing placental single RNAseq dataset^63^, the bulk RNAseq data for each sample was deconvoluted to estimate placental cell type proportions for stromal, Hofbauer, extravillous trophoblast (EVTs), cytotrophoblast, endothelial, and decidual cells. To identify genes differentially expressed by PAE status (pregnancy-wide and periconceptional), we performed DESeq2^64^, adjusting for 7 surrogate variables, gestational age, infant sex, maternal age, and 6 cell type proportions. For weighted gene co-expression network analysis (WGCNA)^65^, the surrogate variables were regressed out following variance stabilization transformation of the data prior to input for the WGCNA workflow, as recommended^66^. We constructed an unsigned network using a soft threshold power = 6, which fit a scale-free network topology with an R^2 = 0.95. Similar modules were merged based on a tree cut height 0.45, yielding 19 modules. Genes that did not uniquely map to specific modules (n = 197) were not included in the network analysis. The first principal component of each module, the module eigengene, was used as a summary measure of each module. The biologic processes of the Gene Ontology database were queried using the enrichR package^67^ to assign biological processes enriched among the modules. Hub genes were defined as the top 15 genes based on the intramodular connectivity score. PAE-related modules were identified based on enrichment for alcohol-associated differentially expressed genes using Fisher’s exact tests. Associations between module eigengenes and PAE variables were also examined based on Pearson correlations and generalized linear models adjusted for 6 cell types, gestational age, infant sex, and maternal age. All analyses were conducted using R version 4.1.1. The code implemented to generate the presented results is located here: https://github.com/Deyssenroth-Lab/SA-FASD-RNAseq.

### Supplementary Information

Supplementary Figure 1.

Supplementary Table 1.

## Data Availability

Derived, deidentified data supporting the findings of this study are available from the corresponding author on request.
