# Supplementary material for: Prenatal alcohol exposure is associated with changes in placental gene co-expression networks
Source: Sci Rep. 2024 Feb 1;14:2687. doi: 10.1038/s41598-024-52737-6 (PMC10834523; doi:10.1038/s41598-024-52737-6)
Supplement: Supplementary file 1 — Supplementary Figure 1. [file 41598_2024_52737_MOESM1_ESM.pdf]

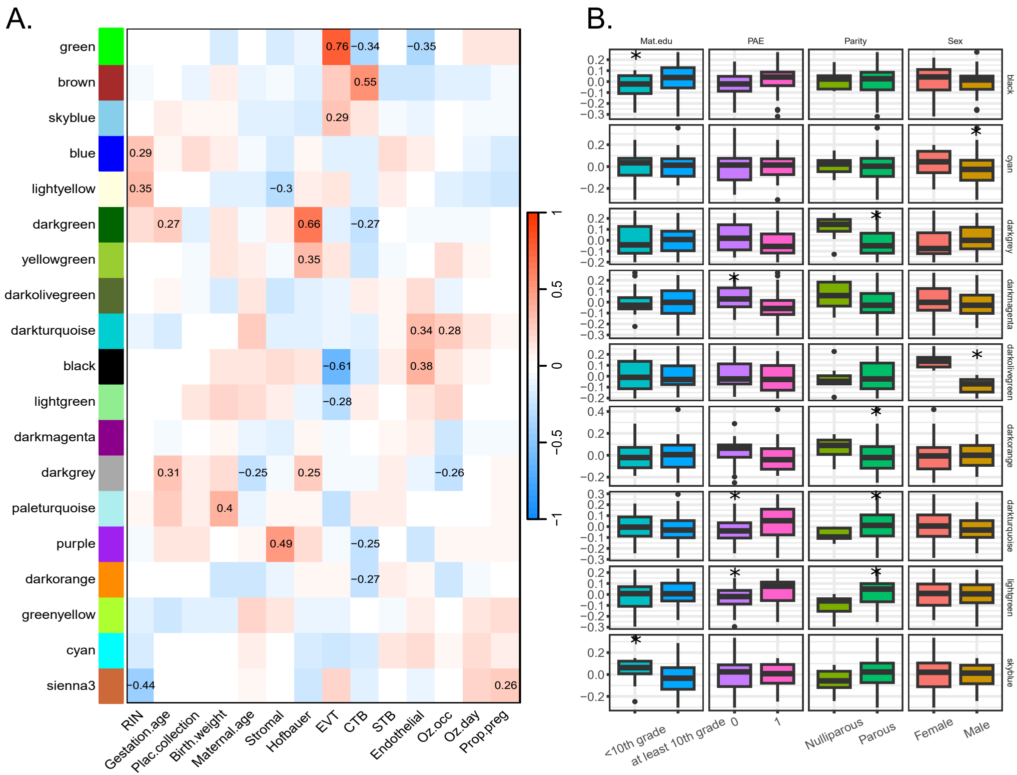

Supplemental Figure 1. Module eigengene relationships with participant characteristics. A. Spearman correlation coeffiecients are shown for significant associations (p<0.05). Color gradient indicates posititve (blue) to negative (red) associations. B. Significant differences in module eigengene values across categorical variables are indicated by an asterisk (*).
